# Mammals fail to regenerate organs when wound contraction drives scar formation

**DOI:** 10.1038/s41536-021-00149-9

**Published:** 2021-07-22

**Authors:** Ioannis V. Yannas, Dimitrios S. Tzeranis

**Affiliations:** 1 Department of Mechanical Engineering, Massachusetts Institute of Technology, Cambridge, MA USA; 2 Department of Mechanical and Manufacturing Engineering, University of Cyprus, Nicosia, Cyprus

**Keywords:** Regenerative medicine, Trauma

## Abstract

To understand why mammals generally do not regenerate injured organs, we considered the exceptional case of spontaneous skin regeneration in the early lamb fetus. Whereas during the early fetal stage skin wounds heal by regeneration, in the late fetal stage, and after birth, skin wounds close instead by scar formation. We review independent evidence that this switch in wound healing response coincides with the onset of wound contraction, which is also enabled during late fetal gestation. The crucial role of wound contraction in determining the wound healing outcome in adults has been demonstrated in three mammalian models of severe injury (excised guinea pig skin, transected rat sciatic nerve, excised rabbit conjunctival stroma) where grafting the injury with DRT, a contraction-blocking scaffold of highly-specific structure, altered significantly the wound healing outcome. While spontaneous healing resulted in scar formation in these animal models, DRT grafting significantly reduced the extent of wound contraction, prevented scar synthesis, and resulted in partial regeneration. These findings, as well as independent data from species that heal spontaneously via regeneration, point to a striking hypothesis: The process of regeneration lies dormant in mammals until appropriately activated by injury. In spontaneous wound healing of the late fetus and in adult mammals, wound contraction impedes such endogenous regeneration mechanisms. However, engineered treatments, such as DRT, that block wound contraction can cancel its effects and favor wound healing by regeneration instead of scar formation.

## Introduction

Mammals in their early fetal stage have been shown capable of regenerating skin following severe injury^1,2^. Investigators have been impressed by fetal wound healing observed in several mammalian species, and have occasionally referred to it as “flawless” while others have described the process as “resembling regeneration”. However, at about two-thirds of gestation time (late fetal stage) the mammalian fetus experiences a transition from scarless skin wound healing to healing that leads to scar formation^3–6^. Beyond that fetal transition, and for the remainder of the organism’s life, skin wounds heal in the standard adult mode, i.e. by scar formation; spontaneous regeneration of adult skin is not observed. With very few exceptions, e.g. rabbit ears^7^ or children’s digits^8^, adult mammals fail to spontaneously regenerate skin or any of their injured organs. In sharp contrast to the late mammalian fetus or adult mammals, several amphibians can regenerate entire limbs spontaneously throughout their lifetime^9^.

Investigators have tried for many years to understand how the early mammalian fetus heals without scar in order to apply this knowledge towards functional restoration in diseased organs in humans^1–3,10^. However, the reason behind the loss of regenerative ability during gestation remains unknown. Today it is widely believed that the ability to spontaneously regenerate limbs or organs is an intrinsic property of mammals that cannot be changed later in life.

This article summarizes experimental evidence from fetal wound healing as well as from three adult mammalian injury models where partial organ regeneration was induced by scaffolds of highly specific properties. Evidence is also marshaled from exceptional mammalian species that can spontaneously heal injuries via regeneration in the absence of wound contraction. Combined together, the evidence suggests a key role for wound contraction in impeding spontaneous regeneration and leading instead to scar formation^11^. We hypothesize that the early fetal competence to heal without scar lies dormant in adult mammals and, once reactivated by proper treatments that block wound contraction, can induce regeneration in adult injured or diseased organs.

## Wound contraction is enabled during gestation

In adult mammals, it is widely recognized that the normal macroscopic forces of wound contraction in skin injuries are generated by contractile cells including myofibroblasts (MFB)^12,13^. Indeed, immunostaining for alpha-smooth muscle actin (αSMA; major MFB marker) appears in adult rat skin wounds about 4 days after injury and disappears, due to MFB apoptosis, about 25 days later^14^.

Apart from adult wounds, MFBs appear to play a significant contractile role in fetal wounds. Motivated by the earlier discovery that fetal skin wound healing switches from scarless healing to scar formation during gestation, Estes and coworkers asked whether this transition was related to the extent of MFB differentiation observed in fetal wounds^15^. Indeed, a significant increase in αSMA staining was observed in fetal lambs upon the transition from early to late gestation, indicative of increased MFB content. αSMA staining was absent in wounds generated at gestation day 75 but was present at progressively greater amounts in wounds generated at gestation day 100 or 120 (term, 145 days). Another convincing demonstration that MFB is associated with wound contraction and scar formation in fetal wounds was provided by Cass and coworkers^16^. This study confirmed that the healing of fetal lamb skin wounds by regeneration was gradually abolished when injury generation was delayed from gestational day 60–90, while simultaneously scar formation progressively dominated wound healing. The observations of Estes et al. and Cass et al. can be explained by the simple hypothesis that gestation age affects, through some yet unknown mechanism, MFB differentiation, which in turn switches the mode of fetal skin wound healing from regeneration to scar formation.

## Scar formation is a key outcome of wound contraction

**Fig. 1:**
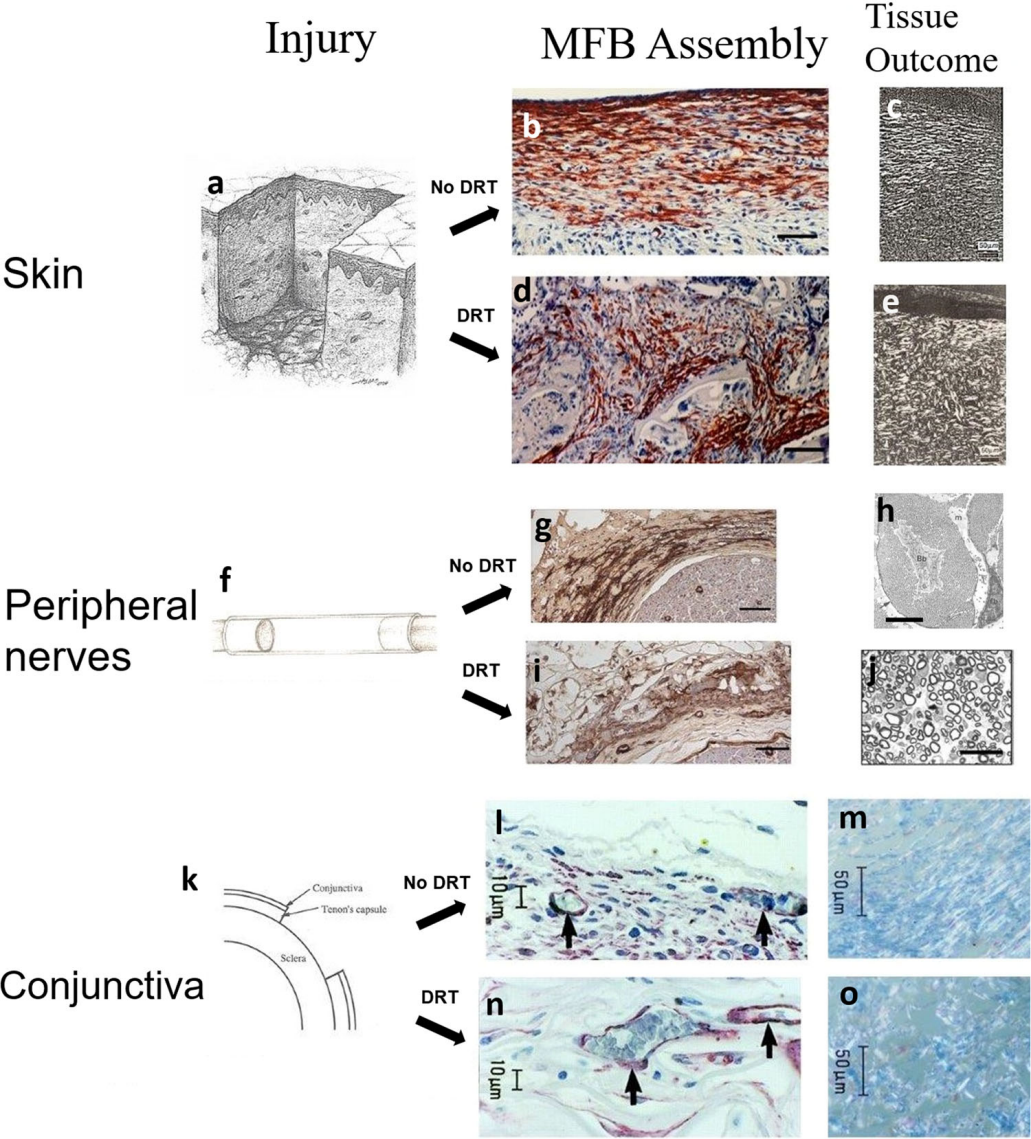
Wound contraction and associated wound healing response in three adult injury models in the absence (spontaneous healing) and presence of a DRT graft. **a**–**e** guinea pig full-thickness excised skin wound. **f**–**j** fully transected rat sciatic nerve. **k**–**o** excised rabbit conjunctival stroma. **a**, **f**, and **k** Schematic of the corresponding injury site model. **b**, **d**, **g**, **i**, **l**, and **n** Immunohistochemical localization of αSMA^+^ myofibroblasts (*red brown*) 10 days after skin injury (**b**, **d**)^20^, 7 days after peripheral nerve transection (**g**, **i**)^20^, or 14 days after conjunctiva injury (**l**, **n**)^27^. **b**, **g**, and **l** Lack of DRT grafting or grafting with control grafts led to large, dense, highly-aligned MFB configurations. **d**, **i**, and **n** DRT grafting led to significantly fewer dispersed, and almost randomly aligned MFBs. Scale bars: skin and nerves, 100 μm; conjunctiva, 10 μm. **c**, **e**, **h**, **j**, **m**, and **o** Evaluating the structure of the resulting tissue. **c**, **e** In full-thickness skin wounds birefringence microscopy of collagen fibers demonstrates the formation of scar in ungrafted wounds (**c**) and the synthesis of the nearly-physiological dermis in DRT-grafted wounds (**e**)^18,26,30^. **h** In DRT-ungrafted peripheral nerve wounds electron microscopy reveals that 26 months following transection, the original nerve fibers have been replaced by a dense sheaf of collagen fibrils that enclose groups of Schwann cells (Büngner bands, Bb)^32^. **j** In contrast, histological micrographs of cross-sections from DRT-grafted peripheral nerve wounds demonstrate the formation of neural tissue whose histomorphometric (equivalent diameter, number of myelinated fibers, number of A-fibers) and electrophysiological assays were similar to those for the autograft^31^. **m**, **o** In conjunctiva wounds, immunohistochemical analysis and birefringence microcopy demonstrate scar formation in ungrafted wounds (**m**, note marked orientation of birefringent collagen fibers) and synthesis of near-normal conjunctival stroma collagen fibers (absence of orientation) in DRT-grafted wounds (**o**)^27^. Scale bars: skin, 50 μm; peripheral nerves, neural scar (top), 1 μm; peripheral nerve, regenerated nerve (bottom), 25 μm; conjunctiva, 50 μm. Figure 1a, f was reproduced with permission from Springer Nature^29^. Fig. 1b, d, g, i, and j were reproduced with permission from Biomaterials, 33, 4783–91, ^©^Elsevier (2012). Figure 1c, e were reproduced with permission from MIT^30^. Figure 1h was reproduced with permission from J. Anat., 192, 529–39, ^©^Wiley (1998). Figure 1l–o was reproduced with permission from Invest. Ophthalmol. Vis. Sci, 41, 2404–11, ^©^Association for Research in Vision and Ophthalmology (2000).

Scar synthesis, the hallmark of failed regeneration in injured skin, has been widely studied in adult mammals; in contrast, the study of wound contraction has been relatively neglected. In a contracting adult skin wound, large numbers of MFBs are held together in assemblies by intercellular junctions^13^. The organization of such MFB assemblies, particularly the spatial orientation of individual MFB axes, appear to be aligned with the physiological macroscopic wound contraction forces that eventually reduce wound size^17,18^. The spatial distribution of MFB assemblies and the resulting mechanical stress field generated in the injury site due to the contractile forces applied by MFB depend on the anatomical shape of the injured organ and the local geometry of the wound undergoing healing. For example, skin wounds (planar geometry) in rodents close by stresses aligned along the epidermal plane; wounded stumps that result from transection of the rat sciatic nerve (cylindrical geometry) close by circumferential compressive stresses (hoop stresses) (Fig. 1b, g)^19,20^. It has been shown that collagen-synthesizing cells, such as fibroblasts and MFBs, deposit newly-synthesized collagen fibers along a direction approximately parallel to their own axes^21^. Accordingly, the average orientation of collagen fibers synthesized during spontaneous wound healing coincides with the major direction of the mechanical stress field generated by highly-organized MFB clusters, leading to the synthesis of a highly anisotropic fibrous tissue (scar)^22–24^. Furthermore, experiments with full-thickness skin wounds in the guinea pig^18^ and in the transected rat sciatic nerve^19^ have shown that wound contraction precedes scar formation by several days^11^.

We hypothesize that scar formation is driven by wound contraction according to the following sequence: Tissue injury generates a wound, which normally closes by a mechanical contraction force. This force acts on the fluid and cellular contents of the wound, orienting the axes of myofibroblasts, as shown in the data for wounds for three injured organs (Fig. 1b, d, g, i, l, and n). Since it has been shown that fibroblasts synthesize collagen fibrils with fiber axes that are parallel to the long axis of the synthesizing cells^21^, oriented fibroblasts in the wound bed are expected to synthesize collagen fibers that are likewise oriented along the same macroscopic axis. This process leads to the formation of scar tissue, a bundle of highly aligned collagen fibers, as previously observed by scanning electron microscopy^22^ and laser light scattering^23^. Clearly, this hypothetical mechanism relies on the effect of a mechanical force and neglects other factors, such as hypothetical regulation of the inflammatory response, e.g. by modulation of the immune cell milieu, that modifies healing to yield scarring. Previous studies have demonstrated the effect of mechanical forces on collagen synthesis by fibroblasts, similar to the forces discussed here^25^.

## Blocking of wound contraction by DRT

A simple and direct test of the hypothesis that wound contraction is associated with scar formation consists of using an efficient contraction-blocker and observing if such use leads to the prevention of scars. Dermis regeneration template (DRT), a highly porous collagen-based scaffold, has been shown to be effective in blocking contraction in skin wounds^26^, peripheral nerve wounds^20^, and the wounded conjunctival stroma^27^.

The effect of DRT on MFB morphology is profound. Blocking of the macroscopic wound contraction force by DRT is associated with the combined effect of a sharp decrease in MFB density, dispersion of MFB assemblies, and disorientation of MFB long axes. The decrease in MFB density by DRT, down to 20% of untreated controls^18^, appears to follow from the observed DRT-induced downregulation of the inflammatory response^20^, including cytokines required for MFB differentiation such as TGFβ1^12^. Dispersion of MFB assemblies and disorientation of MFB long axes is associated with extensive binding of MFB on the DRT surface^28^. Extensive binding of this sort requires a sufficiently high magnitude of specific surface area inside the porous scaffold, a requirement which is satisfied by a small average pore diameter, about 100 μm in an optimized scaffold^26^.

The surface chemistry (density of ligands for specific integrins) of collagen scaffolds is pivotal for the DRT-MFB binding interaction. It has been shown that MFB cannot attach on collagen scaffolds whose integrin ligands have been chemically modified^28^. Integrin-ligand binding in DRT-treated wounds apparently changes the MFB-MFB binding mode characteristic of MFB assemblies in normally contracting wounds to a mostly MFB-DRT binding mode which keeps MFB apart from each other and disrupts MFB assemblies. Modification of the contractile phenotype is eventually followed by blocking of scar and incidence of regeneration (see below).

A detailed description of methods for preparing DRT scaffolds suitable for grafting wounds in skin and peripheral nerves, as well as further details on the mechanism for DRT activity, have appeared in a monograph^29^.

## Blocking wound contraction in three adult mammalian species leads to partial regeneration

Direct support of the hypothesis that inability to regenerate injured adult organs is associated with the presence of wound contraction is provided in a series of experimental studies, which focused on the property of DRT to block wound contraction and induce regeneration in three models of adult mammalian injury: fully-excised guinea pig skin^11,18,26,30^, transected rat sciatic nerve^19,20,31^, and fully-excised conjunctival stroma^27^.

During spontaneous healing (no DRT grafting) the spatial morphology of MFB in the three injured sites differed in apparent deference to the anatomical wound shape which required closure. MFB was organized in a thick planar layer (skin, conjunctiva) or a circumferential layer (peripheral nerves) of dense highly-organized MFB cells suggesting the occurrence of significant wound contraction that had adapted to the shape of the specific injury and organ (Fig. 1b, g, and l).

In all three animal models, spontaneous healing (no DRT grafting) after severe injury led to scar formation (Fig. 1c, h, and m). Specifically, spontaneous healing of full-thickness skin wounds in the guinea pig led to the formation of highly oriented collagen fibers in the plane of the epidermis, characteristic of dermal scar (Fig. 1c)^30^ nerve fibers in the transected rat sciatic nerve trunk lost their characteristic structure and presented instead of a sheaf of collagen fibers, identified as a neural scar (Fig. 1h)^32^ excisions of the conjunctival stroma in a rabbit led to the formation of fibrous tissue, identified as a scar, that was highly oriented in the plane of the stroma (Fig. 1m)^27^.

In contrast, in all three models, grafting the injury site with DRT greatly reduced the number of MFBs at the injury site, dispersed MFB assemblies, and disoriented MFB axes (Fig. 1d, i, and n). As detailed in the preceding section, each of these MFB phenotypic changes observed in the presence of DRT has been associated with the observed abolition of macroscopic wound contraction. In all three animal models, grafting the injury site with DRT led to the synthesis of physiological or nearly physiological new tissue (Fig. 1e, j, and o). Indeed, the histomorphometric and electrophysiological properties of transected rat sciatic nerves, studied at an initial 10 mm gap between transected stumps that was tubulated with DRT conduits, were statistically indistinguishable from those of the autograft control at 60 weeks post injury^31^.

**Fig. 2:**
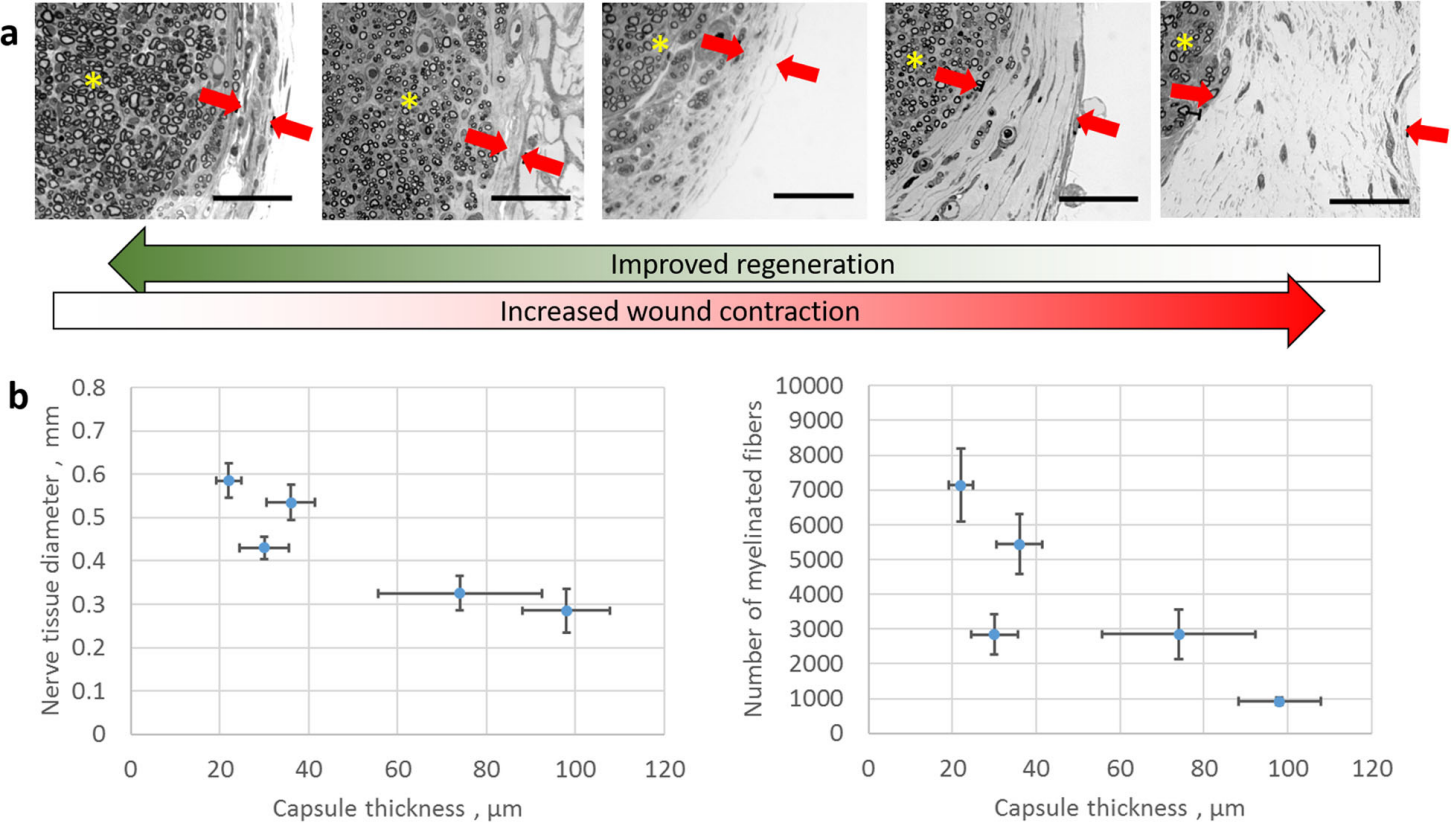
An inverse relationship between wound contraction and induced regeneration was demonstrated in transected rat sciatic nerves grafted by a library of five different porous collagen scaffolds, either DRT or analogs of DRT, differing only in degradation half-life. **a** OsO_4_ staining of tissue sections reveals the formation of a contractile MFB capsule (thickness shown between red arrows) around the newly-formed nerve tissue (stars) 9 weeks post-injury. **b** Quantification of the inverse relationship between the intensity of wound contraction (assayed by the radial thickness of the MFB capsule) and quality of induced regeneration, assayed both by the equivalent tissue diameter (left) and the number of myelinated fibers (right). Data (mean ± se) were obtained at the midpoint of the gap distance, 15 mm, initially separating the two nerve stumps, measured 9 weeks post-injury^20^. Scale bars 50 μm. Figure 2a, b were reproduced with permission from Biomaterials, 33, 4783–91, ^©^Elsevier (2012).

A key observation that supports a hypothetical antagonistic relationship between wound contraction and induced regeneration is the known dependence of the contraction-blocking and partially regenerating activity of DRT grafts on the specific physicochemical properties of the scaffold^26^. In addition to presenting to cells surface-chemical features specific for integrin-ligand binding^28^, DRT scaffolds are further characterized by optimal levels of the degradation half-life and the average pore size^20,26^. Deliberate synthesis of grafts with progressively suboptimal properties resulted in increasingly larger MFB capsules in peripheral nerve stumps and gradually resulted in worse wound healing outcomes that eventually inclined towards spontaneous healing. In particular, a DRT scaffold library, synthesized by systematically perturbing specific DRT properties away from their optimal values, led to scaffolds that were gradually less active regeneratively than DRT^20^. Quantitative data from transected rat peripheral nerves grafted with scaffolds from such a DRT library highlight an inverse relationship between the thickness of the contractile MFB capsule surrounding the transected nerve stump (an indirect assay of wound contraction magnitude) and two metrics of the quality of the resulting nerve tissue (regenerated nerve diameter and number of myelinated axons)^20^, as presented in Fig. 2. The data in Fig, 2 represent direct, quantitative support of the antagonistic relation between contraction and induced regeneration of peripheral nerves.

In summary, experimental data from three adult mammalian models of severe injury (Figs. 1, 2) provide multifaceted support of the hypothesis that blocking of wound contraction by DRT prevents scar formation and favors regeneration.

## Discussion

While the early fetus has been shown capable of regenerating severe skin wounds, the late fetus and the adult mammal spontaneously close skin wounds by contraction and scar formation rather than regeneration. Yet, the observed ability, reported here, to induce partial regeneration in three adult mammalian injury models by DRT grafts that blocked wound contraction (Figs. 1, 2) suggests that the late fetus and the adult mammal retain the ability to regenerate their injured organs if healing is properly guided.

The observation that the occurrence of wound contraction consistently leads to healing by scar formation in adult mammals supports the hypothesis that wound contraction prevents the synthesis of normal organ tissue (regeneration). This hypothesis as well as its reverse (blocking of wound contraction prevents scar formation and favors regeneration) are directly supported by the observed ability of DRT grafts to block wound contraction and its hypothetical sequel, scar formation, and instead induce regeneration in the three animal organ injury models discussed above (Fig. 1). This hypothesis is directly supported by the observed inverse relation between the thickness of the contractile cell capsule and each of two measures of quality of nerve regeneration (Fig. 2).

**Fig. 3:**
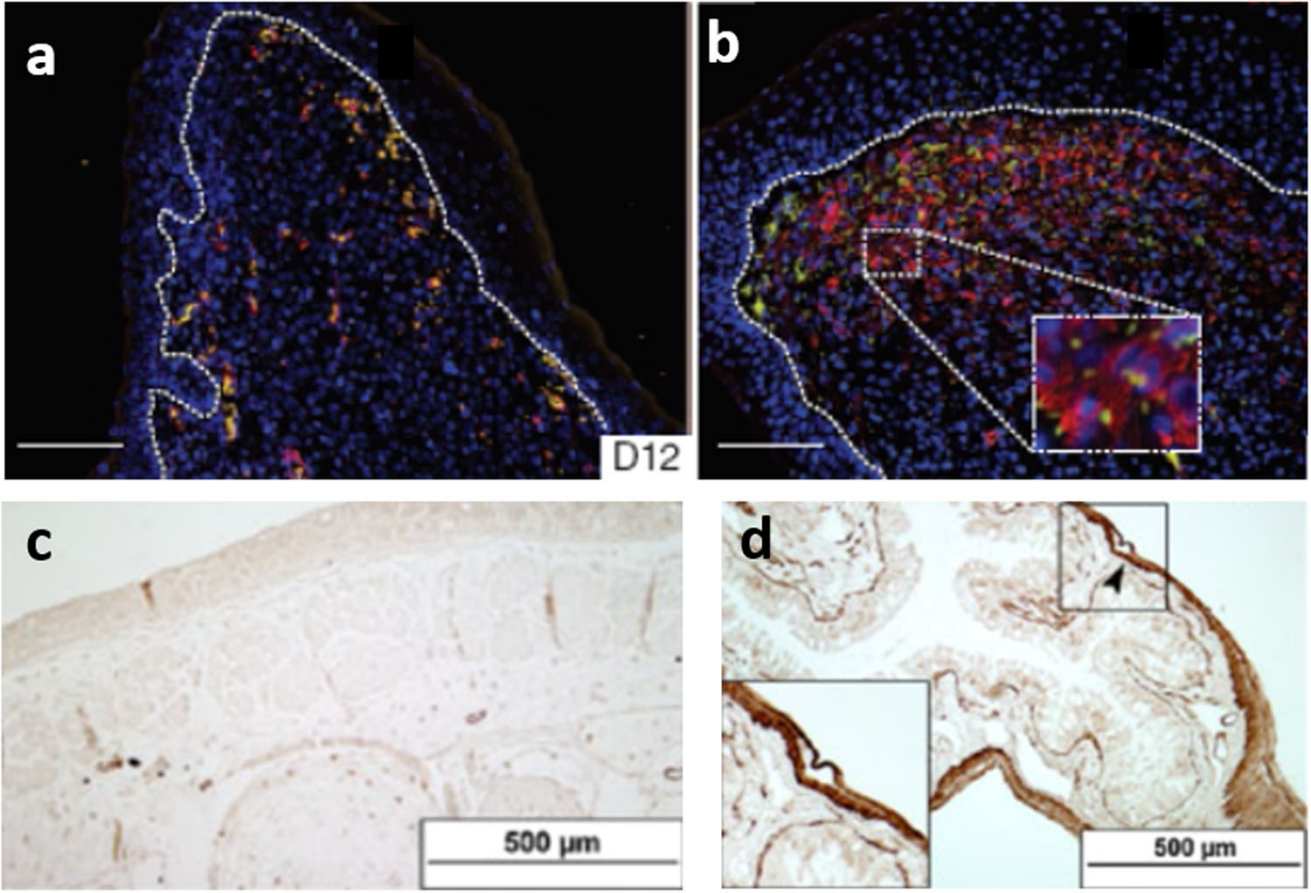
The absence of wound contraction has been reported in several cases of spontaneous scarless healing in various species. **a** No αSMA^+^ fibroblasts (red) were detected 12 days after full-thickness ear injury in *A. kempi*, which are able to spontaneously regenerate severe skin injuries. **b** In contrast, a significant number of αSMA^+^ cells (red) were detected in ear injuries in *Mus musculus* mice, which spontaneously heal such injuries by forming a scar^35^. **c**, **d** The axolotl can regenerate spontaneously injuries in several organs, including its limbs and tail. **c** αSMA staining was not detected in skin injuries, 12 days after injury. **d** In the same animal, αSMA (brown) was detected in control tissue (small intestine)^37–39^. Figure 3a, b were reproduced with permission from Nature 489, 561–66, ^©^Springer Nature (2012). Figure 3c, d was reproduced with permission from J. Exp. Zool. B. Mol. Dev. Evol., 314B, 684–97, ^©^Wiley (2010).

The unexpected association of induced regeneration with blocked wound contraction is further supported by several independent observations from diverse species. In the well-known case of spontaneous skin regeneration in the injured rabbit ear, direct photographic observation demonstrates the absence of contraction when the skin was excised from one side of the rabbit ear^7,33,34^. Skin injuries in African spiny mice, shown to regenerate spontaneously, contain little αSMA immunostaining 12 days post-injury, in sharp contrast to *Mus musculus* mice that do not spontaneously regenerate severe skin injuries, (Fig. 3a, b)^35^. The absence of significant αSMA staining has been also observed in full-thickness excisional skin wounds in the axolotl, which heal spontaneously without a scar, (Fig. 3c, d)^36–39^. Although not proving a causal relation, these observations support the hypothesis that wound contraction and regeneration are processes that compete during the healing of adult mammalian wounds.

Dependence of the contraction-blocking ability of DRT on its physicochemical properties (pore size, degradation rate, surface chemistry)^20,26,28^ suggests that other therapeutic approaches (biomaterials, cells, small molecules), capable of regulating the molecular machinery of wound contraction, could presumably achieve similarly effective regenerative effects. While fetal wound healing data originate from skin injury studies, evidence from adult injury models presented above extends the effects of wound contraction blocking on regeneration beyond skin wounds to other organs where contraction is a major mechanism for wound healing.

We propose the hypothesis that mammals are apparently born with a built-in latent ability to regenerate injured organs, which remains dormant until it is activated by injury. Diverse experimental evidence supports the hypothesis that wound contraction impedes such a latent regenerative activity during spontaneous wound healing both in the late fetus and in adults. However, the ability of specific contraction-blocking grafts based on DRT to induce regeneration suggests that this hypothetical endogenous regenerative potential can be released by treatments that block the cellular and molecular origins of wound contraction. This hypothesis should affect ongoing efforts to design effective regenerative treatments of all kinds (biomaterials, small molecules, cell therapies) for injured or dysfunctional organs. Any emerging regenerative medicine treatments under investigation may fail to activate the intrinsic regenerative capacity of adult mammals unless their design considers ways to control wound contraction.
